# Hepatitis C Virus Increases Occludin Expression via the Upregulation of Adipose Differentiation-Related Protein

**DOI:** 10.1371/journal.pone.0146000

**Published:** 2016-01-05

**Authors:** Emilie Branche, Stéphanie Conzelmann, Clotilde Parisot, Ludmila Bedert, Pierre L. Lévy, Birke Bartosch, Sophie Clément, Francesco Negro

**Affiliations:** 1 Department of Immunology and Pathology, Faculty of Medicine, Geneva, Switzerland; 2 CRCL, INSERM U1052, CNRS 5286, University of Lyon, Lyon, France; 3 Divisions of Clinical Pathology, University Hospital, Geneva, Switzerland; 4 Gastroenterology and Hepatology, University Hospital, Geneva, Switzerland; SAINT LOUIS UNIVERSITY, UNITED STATES

## Abstract

The hepatitis C virus (HCV) life cycle is closely associated with lipid metabolism. In particular, HCV assembly initiates at the surface of lipid droplets. To further understand the role of lipid droplets in HCV life cycle, we assessed the relationship between HCV and the adipose differentiation-related protein (ADRP), a lipid droplet-associated protein. Different steps of HCV life cycle were assessed in HCV-infected human Huh-7 hepatoma cells overexpressing ADRP upon transduction with a lentiviral vector. HCV infection increased ADRP mRNA and protein expression levels by 2- and 1.5-fold, respectively. The overexpression of ADRP led to an increase of (i) the surface of lipid droplets, (ii) the total cellular neutral lipid content (2.5- and 5-fold increase of triglycerides and cholesterol esters, respectively), (iii) the cellular free cholesterol level (5-fold) and (iv) the HCV particle production and infectivity (by 2- and 3.5-fold, respectively). The investigation of different steps of the HCV life cycle indicated that the ADRP overexpression, while not affecting the viral replication, promoted both virion egress and entry (~12-fold), the latter possibly *via* an increase of its receptor occludin. Moreover, HCV infection induces an increase of both ADRP and occludin expression. In HCV infected cells, the occludin upregulation was fully prevented by the ADRP silencing, suggesting a specific, ADRP-dependent mechanism. Finally, in HCV-infected human livers, occludin and ADRP mRNA expression levels correlated with each other. Alltogether, these findings show that HCV induces ADRP, which in turns appears to confer a favorable environment to viral spread.

## Introduction

Hepatitis C virus (HCV) infection is a major causative agent of acute and chronic liver diseases infecting more than 170 million individuals worldwide. Persistently infected individuals are at risk of progressive liver damage characterized by fibrosis, cirrhosis and hepatocellular carcinoma. The rate of progression seems to depend on many cofactors, such as age at infection, sex, and numerous host genetic variants [1].

HCV infection has been shown to be tightly associated with lipid metabolism. Lipids play an important role at several steps of the HCV life cycle including the viral entry, repliation, assembly and secretion. HCV infectious particles circulate in serum as lipoviroparticles. Their density are very heterogenous and depend on whether particles are associated with low density lipoprotein (LDL) or very low density lipoprotein (VLDL) [2]. Then, HCV particles use receptors implicated in lipid uptake such as LDL-receptor (LDL-R), scavenger receptor class B type I (SR-B1) and Niemann-Pick C1-like 1 (NPC1) to enter into host cells [3–5]. Furthermore, geranylgeranyl lipids generated during the cholesterol synthesis pathway play a crucial role in HCV life cycle, as NS5A needs to bind geranylgeranylated F-box and leucine rich repeat protein 2, in order to promote an efficient viral replication [6]. Most importantly, the HCV core protein localizes at the surface of lipid droplets (LDs) and the assembly of viral particles starts from this critical interaction [7].

LDs consist of an organic core comprised of neutral lipids, mainly triglycerides (TG) and cholesterol esters (CE), surrounded by a monolayer of phospholipids. Some proteins are implicated in the maturation of LDs (such as seipin [8]), while others are structurally associated with LDs. The best characterized LD-associated proteins belong to the PAT [Perilipin (PLIN), ADRP and TIP47] protein family. Tail-interacting protein of 47 kD (TIP47) has been shown to play an important role in HCV life cycle [9, 10]. Another member of the family, PLIN2 or adipophilin/adipocyte differentiation–related protein (ADRP) encoded by *PLIN2/ADRP*, is ubiquitously expressed, including in both normal and steatotic liver [11]. Even if its role is unclear, it may limit the interaction of adipose triglyceride lipase (ATGL) with the neutral lipids within droplets, thus promoting triglyceride accumulation by reducing their turnover [12]. Of note, ATGL has been recently identified as a cofactor of HCV core-induced steatosis [13]. Concerning ADRP, a correlation between its protein expression and steatosis scores has been demonstrated in the liver of nonalcoholic fatty liver disease patients [11] and, even more importantly in our context, its expression is increased in the liver of HCV infected patients when compared to uninfected controls [11].

As HCV can modulate the lipid metabolism, we aimed at determining whether HCV has an impact on ADRP and *vice versa*. We hypothesized that this particular LD-associated protein may play a role in HCV life cycle. Our results suggest that HCV increases ADRP expression which in turn seems to be beneficial to HCV spread.

## Materials and Methods

### Reagents, Antibodies, Plasmids, Primers, and Small Interfering RNAs (siRNAs)

All reagents, antibodies plasmids, primers, and siRNAs used in this study are described in S1 Table.

### Cell Culture

Human embryonic kidney (HEK) 293T and human hepatoma (Huh-7) cells [14] were cultured in low glucose Dulbecco’s modified Eagle’s medium (DMEM) supplemented with 10% fetal bovine serum (FBS), 100 U/ml penicillin, 100 U/ml streptomycin, and 2 mM L-glutamine (all from Invitrogen Life Technologies). Huh-7.5 cells [14] were cultured in complete high glucose GlutaMAX DMEM.

### ADRP expressing lentiviral vector production

ADRP-encoding sequence was subcloned in the gateway lentiviral system (Invitrogen) using the OTB7-ADRP plasmid as template (Openbiosystem) and lentivector particles were produced as previously described [15]. Viral titer was estimated by RT-qPCR [16]. Green fluorescent protein (GFP)-encoding lentivectors were used as controls.

### Silencing of ADRP mRNA

Eighty percent confluent Huh-7 cells were transfected with 5nM ADRP siRNA (Qiagen SI02780043) or 5nM control siRNA (Cell Signaling 6568) or without siRNA using lipofectamine 2000 (Invitrogen), according to the manufacturer’s instructions. After 6h, the medium was changed and cells were incubated for 72h prior to further analysis.

### HCV production, titration, and infection and transient transfection

Chimeric JFH1-J6 (Jc1 [17]) full length RNA was generated from pFK-J6/C3 (Jc1) (from R. Bartenschlager, Heidelberg, Germany), by using the T7 Ribo Max Express Large scale RNA Production System (Promega). *In vitro* transcripts were purified using the Nucleospin RNAII kit (Macherey Nagel), and their integrity was verified with the Agilent 2100 bioanalyzer**.** To assess the level of expression of ADRP and occludin in presence of HCV, Huh-7 cells were transfected 24 hr after plating with 3 μg HCV RNA and 5 μl Lipofectamine 2000. After 48 hr, cells were fixed or harvested for protein or RNA extraction. To produce infectious particles, Huh-7.5 cells (4 × 10^6^) were electroporated with 5 μg of HCV RNA using Amaxa cell line nucleofector kit T (260 V, 950 μF Lonza). Culture supernatant was harvested after 48 h, filtered through 0.45 μm pore-sized polyvinylidene difluoride membrane, and titered by infecting naive Huh-7.5 cells by serial dilutions. Cells were fixed after 48–72 h with −20°C methanol and immunostained using an anti-HCV core (C7-50) antibody. Tissue culture 50% infectious dose (TCID_50_/ml) was calculated as reported [17]. Huh-7 cells were routinely infected with 1 MOI of HCV for 48 h.

To assess intracellular particle infectivity, cells were collected in PBS, counted and lysed by four freeze-thaw cycles in dry ice and a 37°C water bath. After centrifugation, the supernatants were collected and used to determine the particle infectivity by infecting naive Huh-7.5 and calculating TCID_50_/ml.

### RNA isolation, reverse transcription and real-time PCR

Total intracellular RNA was extracted using Trizol (Ambion). Extracellular RNA of HCV particles was extracted with a QIAamp Viral RNA Mini Kit (Qiagen). RNA (1 μg) was used for complementary DNA synthesis with the SuperScript^™^ II RNase H(-) reverse transcriptase (Invitrogen Life Technologies) and random hexanucleotides. For RT-qPCR, the specific primers listed in S1 Table were used. Relative quantification was performed by RT-qPCR as described [18] using eukaryotic translation elongation factor A-1 (eEF1A1) as reference for normalization.

### SDS-PAGE and Immunoblot analyses

Equal amount of proteins from lysed cells were separated by 2–10% SDS-PAGE. Separated proteins were transferred onto a nitrocellulose membrane. After blocking with 5% dry milk, immunoblotting was performed with specific antibodies (S1 Table). Proteins were revealed by chemiluminescence.

### HCV pseudoparticle production

The plasmid phCMV 1b9.9 containing luciferase as a reporter gene was used to produce HCV pseudoparticles based on the method described [19]. Control pseudoparticles were generated with the VSV-G glycoprotein [20]. Luciferase assay was performed 48 h after the transduction using the Dual-Luciferase assay system kit (Promega) according to the manufacturer’s protocol.

### Subgenomic replicon, Transfection and Luciferase reporter gene assay

GFP and ADRP-expressing Huh-7 cell lines were seeded at 200,000 cells per well (6-well plate) and transfected with 2.5 μg of pFK_i389LucNS3 *in vitro* transcript as well as with 0.2 μg pTKrenilla_Luc (kind gift from Dr D. Garcin, Geneva, Switzerland) using 5 μl Lipofectamine 2000 (Invitrogen). Luciferase assay was performed 48 h after the transfection as described above.

### Immunofluorescence and Oil Red O staining

Cells were fixed with 3% paraformaldehyde for 10 min at RT and permeabilized with 0.2% Triton X-100 for 10 min. Cells were first incubated with anti-ADRP in PBS for 1 h at RT, and with Alexa 488 goat anti-mouse (Life Technologies, A11029) and DAPI for 1 h at RT. Neutral lipids were stained 10 min at RT with Oil Red O (ORO). After washing in PBS, cells were mounted in polyvinyl alcohol. Images were acquired using either an Axiophot microscope (Carl Zeiss) equipped with an Axiocam camera (Carl Zeiss) or a confocal microscope (LSM700, Zeiss). Surface area of individual LD was calculated using the Metamorph software (Molecular Devices Corporation).

### Triglyceride,cholesterol ester and free cholesterol measurements

TG and cholesterol were extracted as previously described.[21] TG were measured using the GPO/PAP kit (Roche). CE and free cholesterol were measured using a commercial kit (Calbiochem). TG, CE and free cholesterol were normalized by the protein amounts determined using the BCA protein Assay kit (Pierce).

### HCV infected patient liver samples

We analyzed total RNA extracted from liver biopsy samples taken at the time of routine diagnostic workup in 50 non-diabetic chronic hepatitis C patients with different steatosis scores. Histological data including steatosis scores were recorded using internationally accepted scoring systems [1]. The characteristics of the patients are summarized in Table 1. The protocol was approved by the Ethical Committee of the University Hospitals of Geneva, and all patients gave their informed written consent to the study.

**Table 1:** Baseline features of 50 chronic hepatitis C patients.

Males	43 (86%)
Age (years)^a^	42 (25–69)
BMI^a^	26.6 (19.6–32)^b^
HCV RNA (IU/ml)^a^	2 927 349 (38.10^3^–13.10^6^)^c^
HCV genotype	
1	24 (48%)
2	3 (6%)
3	12 (24%)
4	11 (22%)
Steatosis score^d^:	
0	21 (42%)
1	17 (34%)
2	12 (24%)

^a^, data reported as median values (range)

^b^, values available for 33 patients

^c^, values available for 43 patients

^d^, score defined as follows: 0 = <5% of hepatocytes; 1 = 5–30% of hepatocytes and 2 = >30% of hepatocytes

### Statistical analysis

Results were expressed as mean ± SEM of at least three independent experiments, and analyzed by Student t-test. Values of ***p <0.001, **p <0.01, and *p <0.05 were considered statistically significant.

## Results

### HCV increases ADRP expression

**Fig 1:**
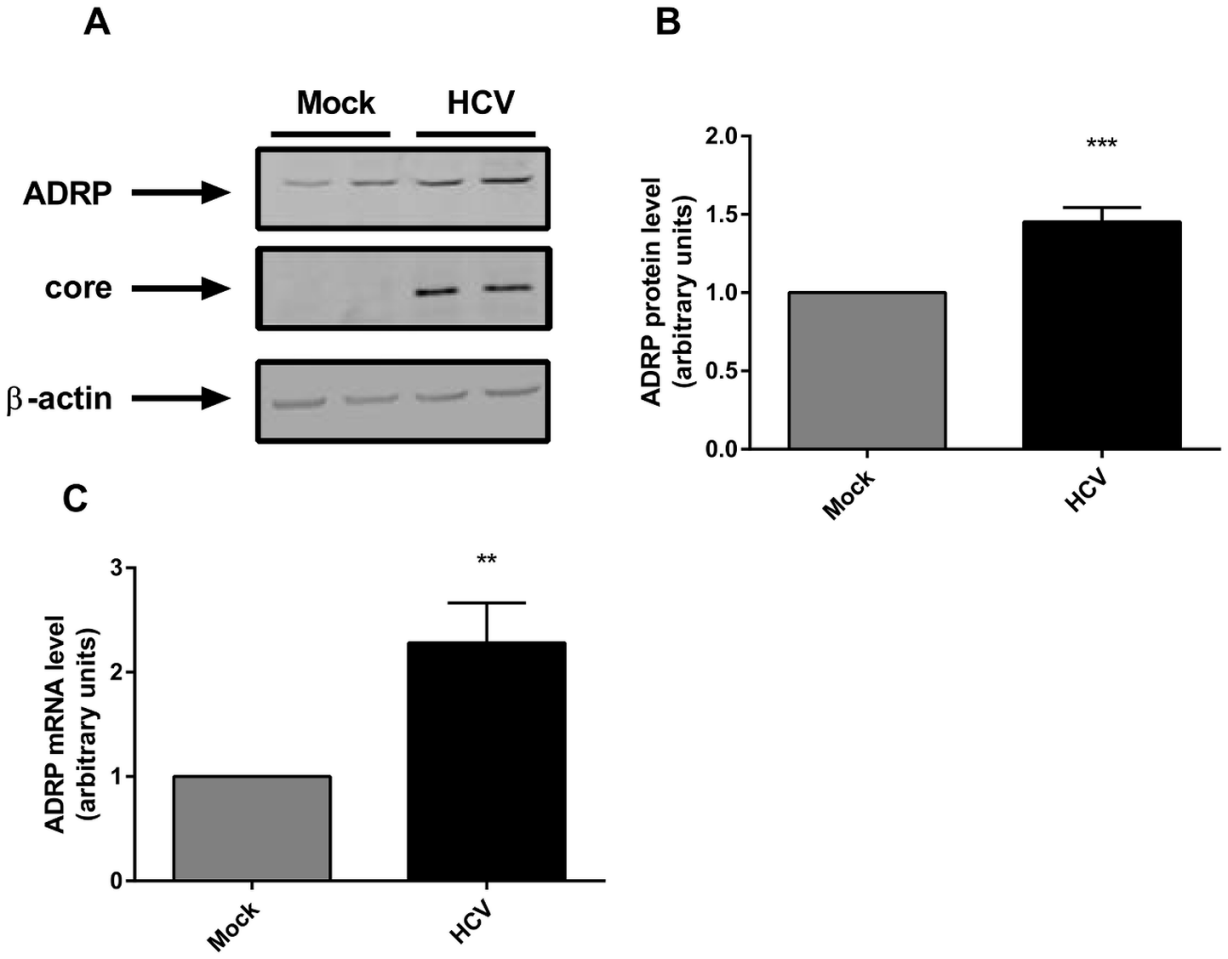
HCV upregulates ADRP expression in Huh-7 cells. **(A)** Representative immunoblot of ADRP, core and β-actin in Huh-7 cells untransfected or transfected with Jc1 full length RNA (HCV). **(B)** Graph represents ADRP protein quantifications of at least three independent experiments. **(C)** ADRP mRNA levels were assessed by RT-qPCR.

As an increase in ADRP had been shown in HCV infected patients [11], we first evaluated whether HCV had a similar effect in our cellular model. To this aim, proteins and mRNAs of Huh-7 cells transfected with Jc1 RNA were harvested 48 hours after transfection. Both ADRP protein and mRNA levels were increased in Jc1 transfected cells compared to untransfected control cells (Fig 1A, 1B and 1C).

### ADRP overexpression increases HCV particle production

**Fig 2:**
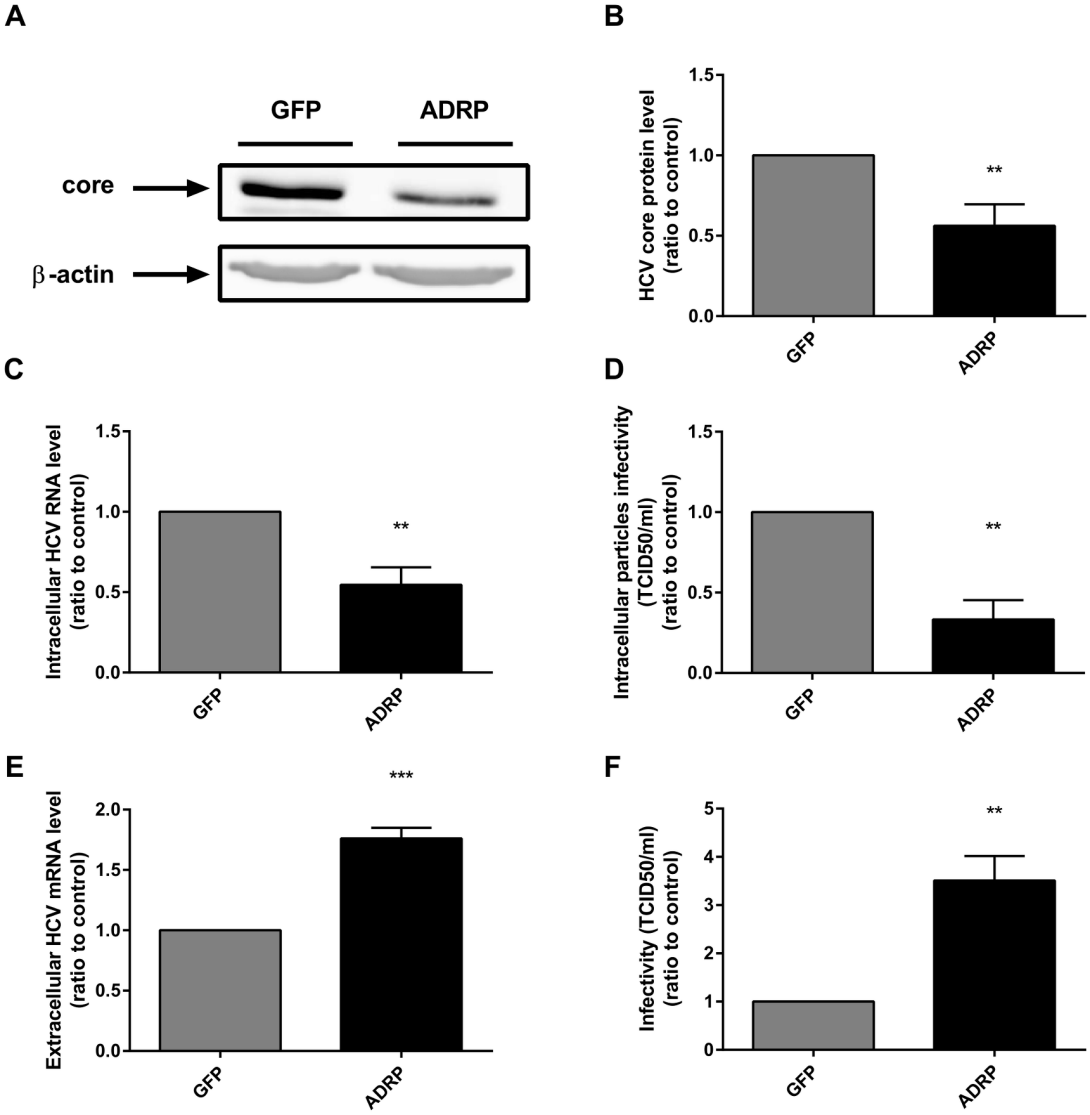
Effect of ADRP overexpression on HCV particle production. GFP and ADRP transduced cells were infected by Jc1 viral particles. Intracellular proteins, RNA and particles were harvested 48 h post infection to assess the level of HCV core protein expression by immunoblot **(A,B)**, the relative number of intracellular HCV RNA copies by RT-qPCR **(C)** and the intracellular particle infectivity by infecting naive Huh-7.5 cells and calculating the TCID/50 **(D)**. In parallel, supernatants were collected to determine the relative number of extracellular HCV RNA copies by RT-qPCR **(E)** and the extracellular particle infectivity by infecting naive Huh-7.5 cells and calculating the TCID_50_/ml **(F).**

**Fig 3:**
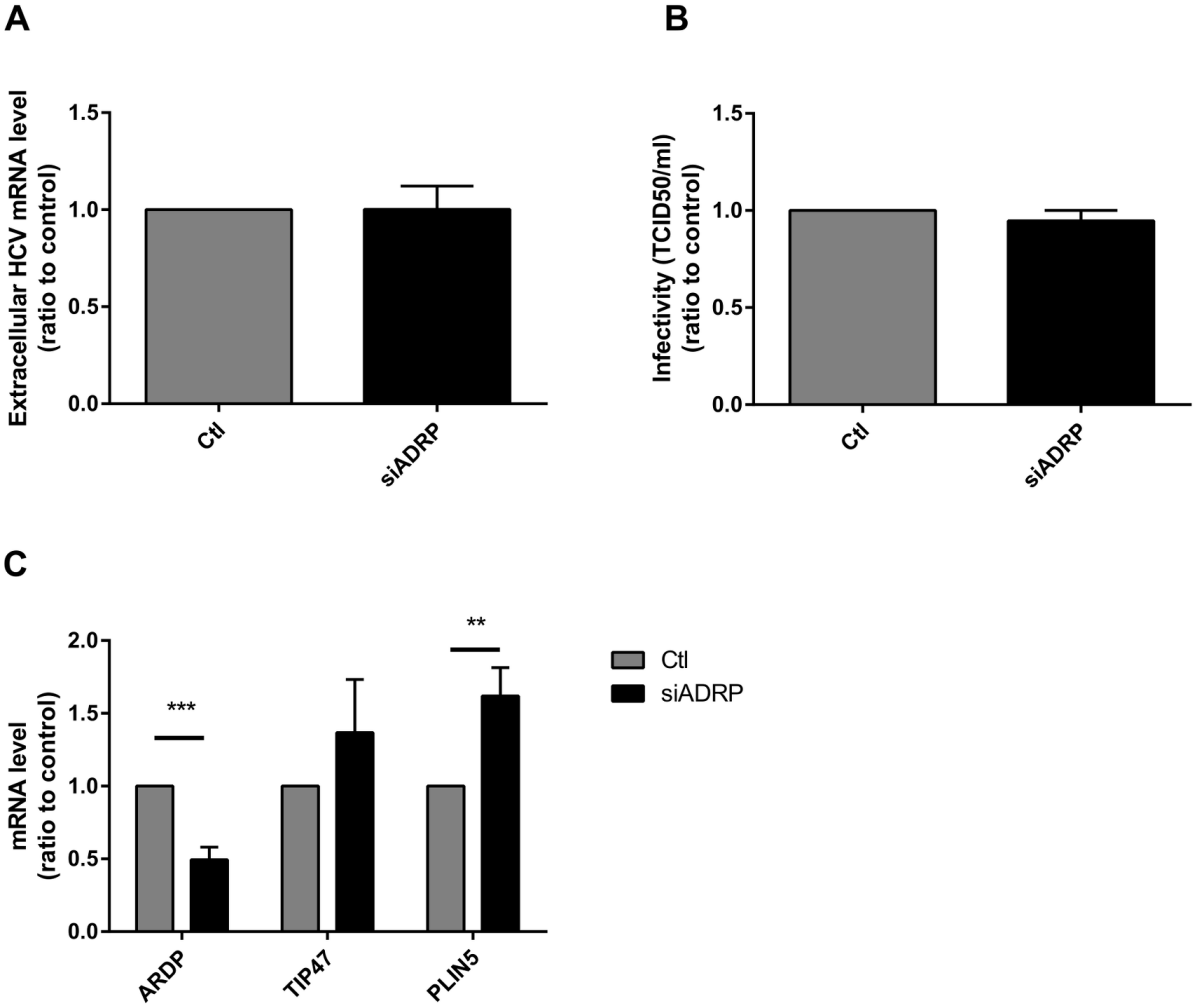
Effect of ADRP silencing on HCV particle production and PAT protein expression. Huh-7 cells were transfected with siRNA targeting ADRP (siADRP). Twenty-four hours post transfection, cells were transfected with HCV Jc1 full length RNA (HCV) for an additional 48 h. Supernatants were then collected to determine the relative number of extracellular HCV RNA copies by RT-qPCR **(A)** and the extracellular particle infectivity by infecting naive Huh-7.5 cells and calculating the TCID/50 **(B)**. ADRP, TIP47 and PLIN5 mRNA levels were determined by RT-qPCR in control or ADRP-silenced Huh-7 cells **(C).**

To further evaluate the implication of ADRP in viral life cycle, we modulated the expression of ADRP by either overexpression or silencing. We first generated ADRP-overexpressing Huh-7 cells, using a lentivector coding for ADRP. Overexpression of ADRP was confirmed at both mRNA and protein levels, 48 hours post transduction (S1A and S1B Fig). As expected, overexpressed ADRP localized around the LDs, as assessed by double staining of ADRP (green) and Oil red O (red). This suggests that ADRP overexpression does not affect its subcellular localization and ADRP even overexpress has the expected localization (S1C Fig). The proper silencing of ADRP by specific siRNA was verified at both RNA and protein levels (S1D, S1E and S1F Fig). The effect of ADRP overexpression on HCV life cycle was evaluated in both control and ADRP overexpressing Huh-7 cells infected with HCV Jc1 particles. ADRP overexpression decreased both intracellular core protein and viral RNA by 40% and 50% respectively, as well as the intracellular particle infectivity by 70% (Fig 2A, 2B, 2C and 2D). However, the number of secreted viral particles (evaluated by HCV RNA quantification in the supernatant), as well as their infectivity (TCID_50_/ml) were increased in ADRP-overexpressing cells when compared to control cells (Fig 2E and 2F). On the contrary, ADRP silencing had no effect on HCV egress (Fig 3A and 3B), which may be explained by a compensation by another member of the PAT family, in this case PLIN5, as reported in other settings (Fig 3C; 1.6-fold increase of PLIN5 mRNA level in ADRP-silenced cells, when compared to control cells).[22, 23]

### ADRP overexpression increases HCV entry *via* an increase of occludin expression

**Fig 4:**
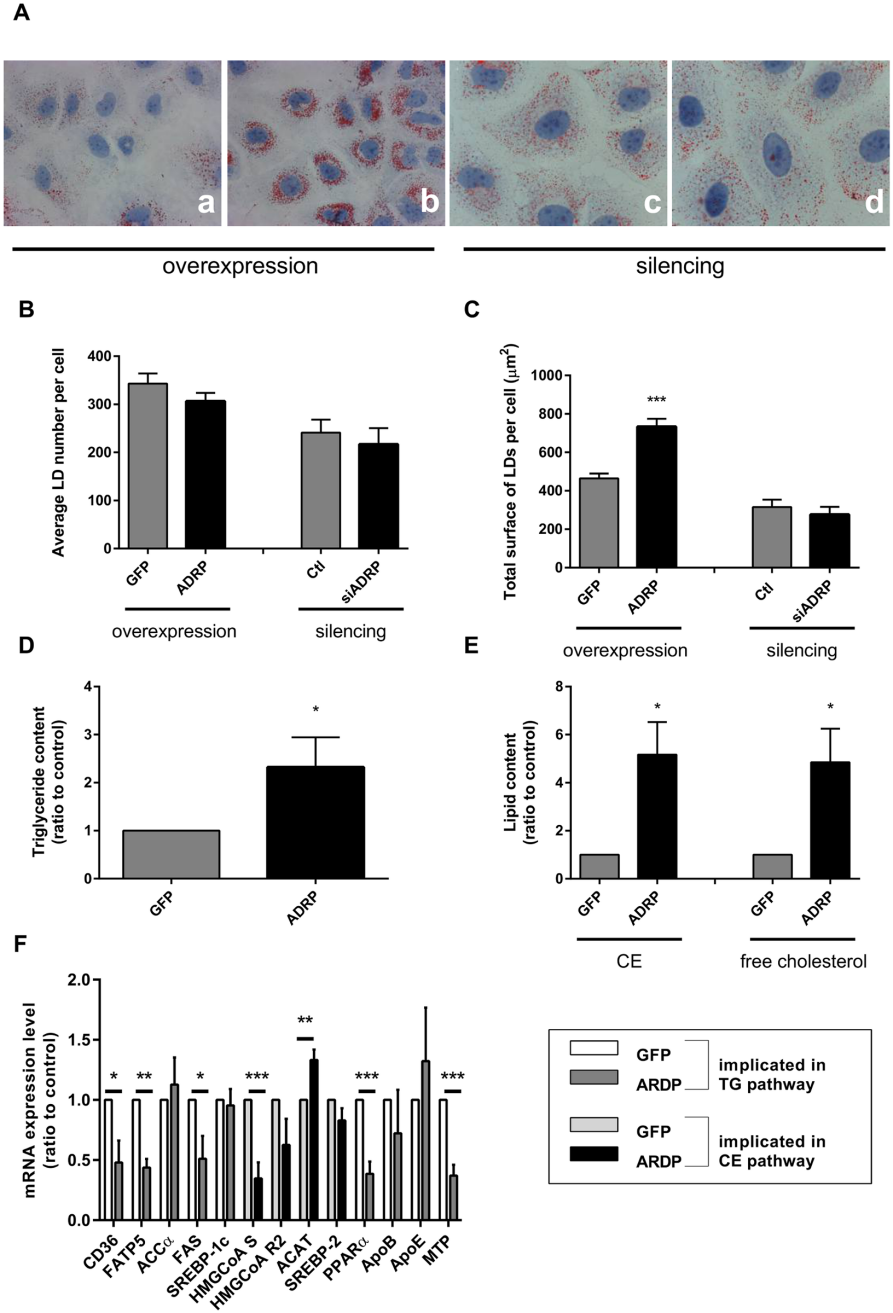
ADRP overexpression, but not ADRP silencing, modifies morphology and lipid content of lipid droplets. **(A)** ORO staining of ADRP overexpressing (b) or silenced (d) cells Huh-7 cells compared to their respective controls (GFP, a and siCont, c). **(B,C)** Determination of morphometric features of LDs in control vs. ADRP overexpressing or silenced cells. LD number per cell **(B)** and total surface of LDs per cell **(C)** were quantified using the Metamorph software. Cellular TG **(D)** and CE and free cholesterol **(E)** contents normalized by the total protein amount are shown. **(F)** mRNA levels of genes implicated in lipid uptake, neosynthesis, β-oxidation and secretion were determined by RT-qPCR.

**Fig 5:**
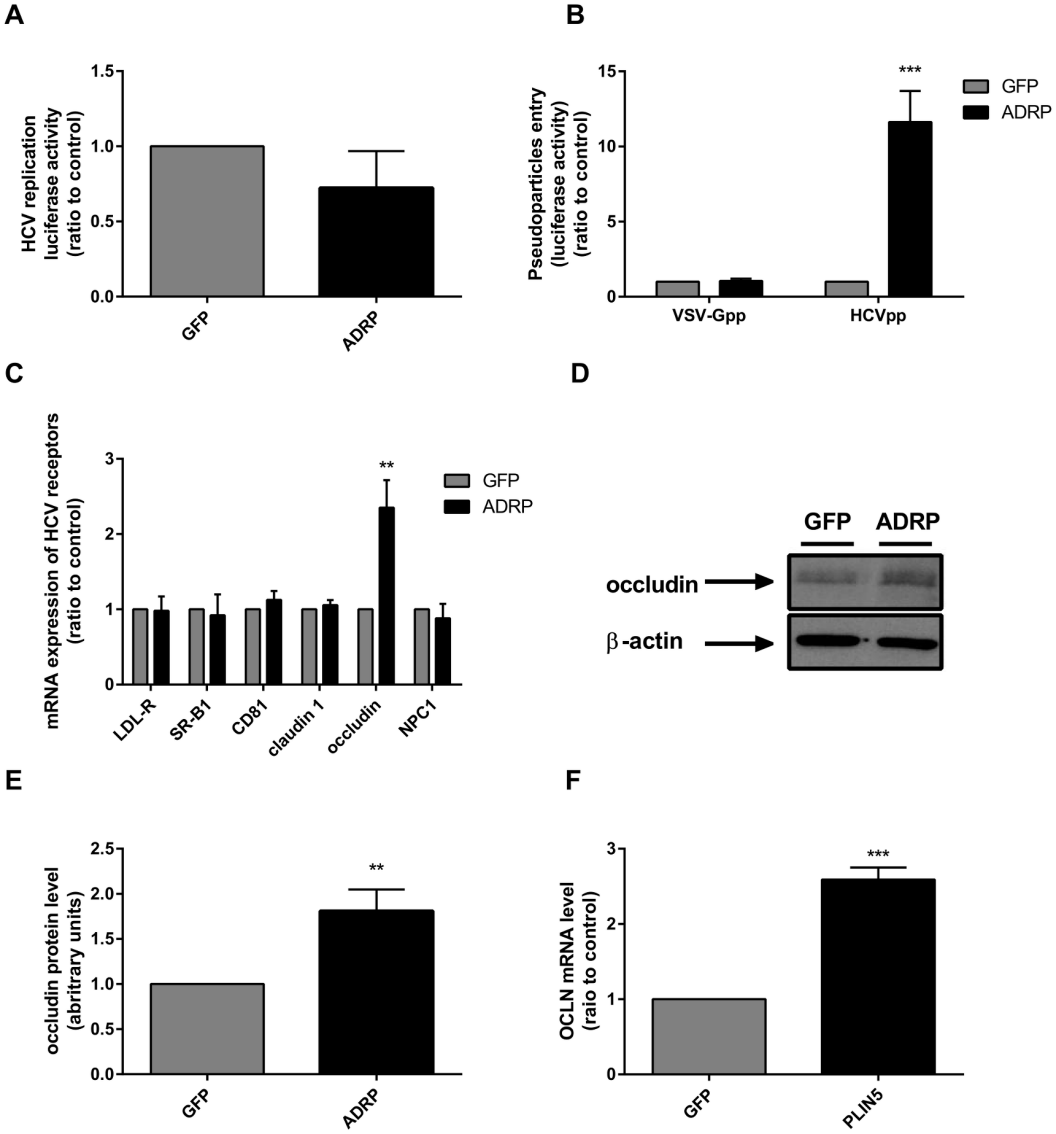
ADRP increases the HCV entry through an upregulation of occludin. **(A)** Control and ADRP-overexpressing cells were transfected with the subgenomic replicon *in vitro* transcript. **(B)** Cells were transduced with HCVpp or VSV-Gpp used as control. After transfection or transduction, luciferase activity was measured by a luciferase assay. **(C)** mRNA levels of HCV receptors were measured by RT-qPCR in control and ADRP-overexpressing cells. **(D and E)** Occludin protein level in GFP or ADRP transduced cells was determined by immunoblotting. **(F)** mRNA level of occludin in GFP and PLIN5 transduced cells was determined by RT-qPCR.

As HCV is believed to assemble at the surface of LDs, we evaluated the effect of ADRP overexpression on LD morphology by ORO staining (Fig 4Aa and 4Ab). Detailed morphometric analyses showed that ADRP overexpression did not affect the LD number per cell (Fig 4B), but induced a 2-fold increase of the total surface of LDs per cell (Fig 4C). In addition, ADRP overexpression induced a significant increase of both TG and CE, the main lipids in the LD core, approximately by 2.5 and 5-fold respectively (Fig 4D and 4E). This indicates that ADRP overexpression increases the surface of LDs per cell through an increase of the lipid content. Additionally, ADRP overexpression induced an 5-fold increase of free cholesterol (Fig 4E). Even if, in fibroblasts [24] and macrophages [25] the ADRP overexpression has been shown to induce lipid accumulation without changing the expression of genes involved in lipid metabolism, we assessed whether this overexpression could have an impact in our hepatic model. Lipid accumulation in LDs can be explained by increased neosynthesis, increased uptake, impaired oxidation, or decreased secretion of lipids. Evaluation by real-time PCR of expression involved in each process allowed to determined which, if any, was affected by ADRP overexpression (Fig 4F). While we observed a decrease of mRNA expression of free fatty acid receptors [CD36 and fatty acid transport protein 5 (FATP5)] (Fig 4F), the expression levels of cholesterol receptors (SR-B1 and LDL-R and NPC1) were unchanged (Fig 5C). As shown in Fig 4F ADRP overexpression likewise induced a decrease of mRNA levels of proteins implicated in lipid neosynthesis, fatty acid synthase (FAS) and 3*-*hydroxy*-*3*-*methyl-glutaryl-CoA (HMGCoA) synthase, possibly caused by a negative feedback. However, the expression of Acetyl-CoA cholesterol acyltransferase (ACAT)—the enzyme converting cholesterol into CE—was increased by 30%. Moreover, we observed a decrease of peroxisome proliferator-activated receptors α, indicating that ADRP overexpression may impact lipid oxidation. Finally, as it is the case for HCV core protein [26], ADRP overexpression decreased the mRNA level of microsomal triglyceride transfer protein, which is required for VLDL secretion**.** In contrast to ADRP overexpression, ADRP silencing did not modify LD morphology (Fig 4Ac, 4Ad, 4B and 4C), consistently with the hypothesis that ADRP silencing is compensated by an increase of PLIN5 expression (Fig 3C).

We then assessed HCV replication and entry using an HCV subgenomic replicon [27] and HCVpp [19], respectively. While ADRP overexpression had no impact on HCV replication (Fig 5A), it increased by 12-fold the HCVpp entry compared to VSV-Gpp entry used as control (Fig 5B). This suggests that ADRP may facilitate HCV entry. Then, we measured HCV receptors following ADRP overexpression, and found that occludin increased at both the mRNA (~2.5-fold) and protein (~2-fold) levels (Fig 5C, 5D and 5E). In accordance with the fact that PLIN5 could compensate the effect of ADRP silencing mentioned above, we observed that also PLIN5 overexpression increased occludin mRNA (Fig 5E).

**Fig 6:**
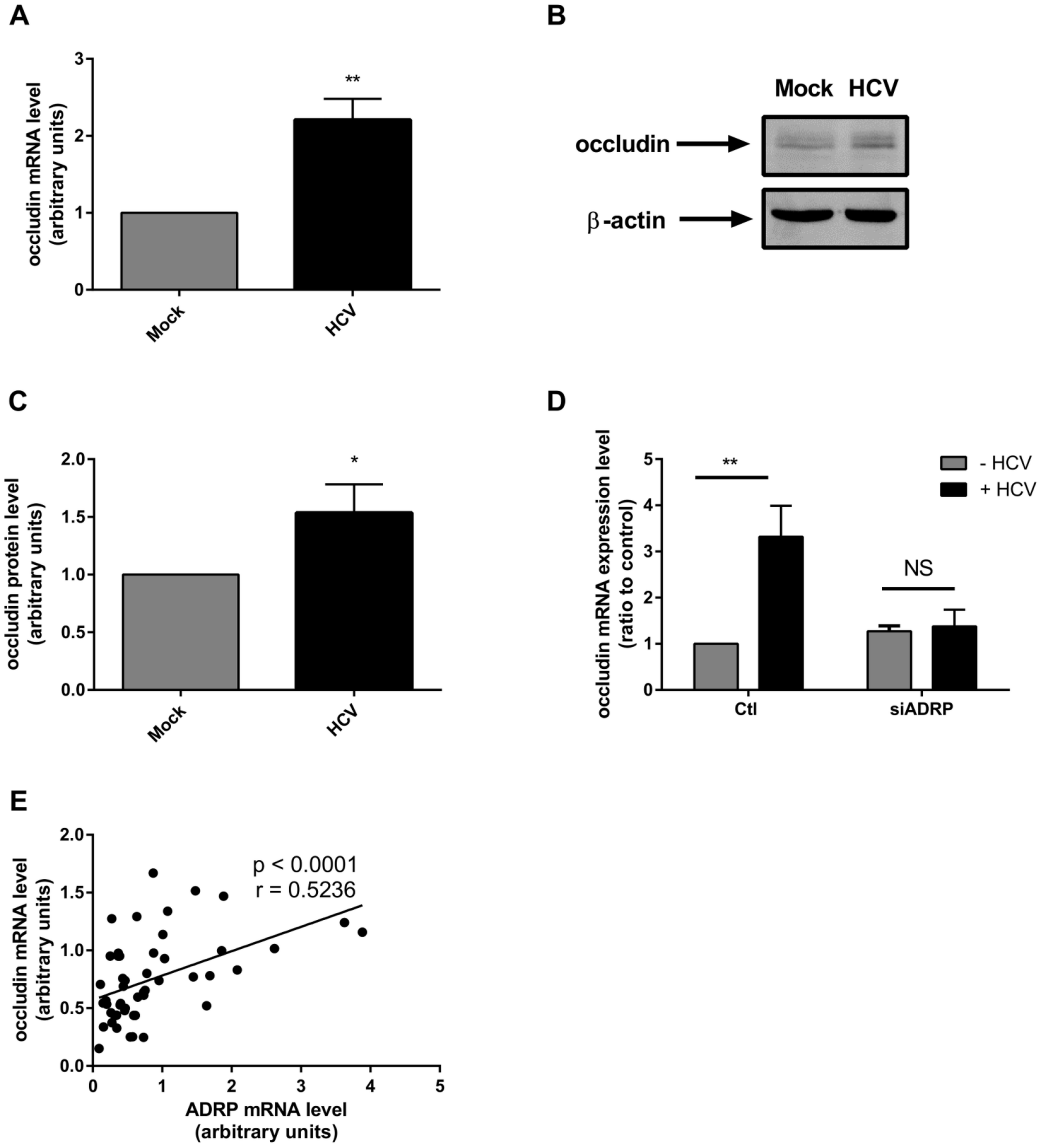
HCV increases occludin expression level *via* a mechanism dependent on ADRP. Huh-7 were transfected with Jc1 RNA (HCV) and occludin mRNA **(A)** and protein **(B-C)** expressions were determined 48 h post transfection by RT-qPCR and immunoblotting respectively. **(D)** Control or ADRP-silenced cells were transfected or not with Jc1 full length RNA (HCV). Forty eight hours post infection, mRNA occludin level was determined by RT-qPCR. **(E)** Correlation between ADRP and occludin mRNA expression level in HCV infected patients (Table 1). Spearman’s r = 0.5236.

Then, since HCV increases ADRP expression *in vitro*, and since ADRP overexpression increases occludin expression, we wondered whether HCV infection would result in an enhanced occludin expression. Since viruses often downregulate their receptors in order to prevent superinfection, one may hypothesize that HCV may have developed a strategy to counteract the ADRP-induced occludin increase. However, as shown in Fig 6A, 6B and 6C, HCV induced an increase of both mRNA and protein levels of occludin. We then hypothesized that HCV-mediated occludin up-regulation might be ADRP-dependent. To challenge this idea, we transfected viral RNA in Huh-7 cells previously silenced for ADRP. Fig 6D shows that HCV–induced occludin up-regulation was fully abrogated by ADRP knockdown, while no effect on occludin expression was observed in uninfected cells. In order to extend our *in vitro* observations to the human infection, we measured the ADRP and occludin mRNA levels in the liver of 50 patients with chronic hepatitis C (Table 1). In infected human livers, a significant correlation between the occludin and ADRP mRNA expression levels was observed **(**Fig 6E**, Spearman r = 0.5236, p<0.0001),** further suggesting that ADRP and occludin increases could be two interconnected phenomena.

## Discussion

We identified ADRP as a new host cell factor implicated in the HCV life cycle. The fact that HCV induces ADRP expression suggests that this particular LD protein may play a role in the establishment of an environment favorable to HCV infection. JFH1 HCV infection has been previously found to reduce the amount of ADRP [28]. This was attributed to core localization around the LDs leading to ADRP displacement and degradation. This discrepancy could partly be explained by the fact that the core proteins of JFH1 and Jc1 display different features subcellular localization. JFH1 core protein appears to be strongly associated to the LD membrane [29, 30], while the core of Jc1—used in our study- is only partially and for a short duration located around the LD [31, 32] and therefore could preserve LD membrane localization of ADRP and prevent its degradation.

Our data suggests that ADRP acts at several non-exclusive levels to promote HCV spread. A first mechanism consists in facilitating the assembly of HCV particles. ADRP overexpression increased the total LD surface without modifying the LD number per cell, resulting in an increase of the total LD membrane surface thought to be the platform for HCV assembly [7]. We recently suggested that a critical factor influencing the efficiency of assembly is the overall LD membrane area available [20]. In fact, the overexpression of the LD fusion protein seipin, leading to a decrease of the total LD membrane area, resulted in a reduced HCV particle production [20]. Second, ADRP overxpression is accompanied by an increase of the total cell content of TG and CE. This is likely to be caused by (i) an increased conversion of cholesterol in CE, (ii) a decrease of lipid oxidation and (iii) a reduced VLDL secretion. The importance of CE in the HCV life cycle, in particular, has been recently emphasized using a potent, specific inhibitor of ACAT which significantly decreased the production of infectious virus without affecting viral RNA replication [33]. In addition, it is noteworthy that ACAT upregulation induced the synthesis of CE providing almost half of the lipids within lipoviroparticles [34]. This suggests that the CE increase induced by ADRP may also promote a more efficient lipoviroparticle secretion. A third mechanism whereby ADRP affects the HCV life cyle involves the virion entry. We observed that ADRP overexpression increased both HCVpp entry and occludin expression. Occludin, the first tight junction integral membrane protein identified [35], is a protein of 60kDa with four transmembrane regions, two extracellular loops and N and C-terminal region projection into the cytoplasm. This protein is located at the epithelial and endothelial tight junctions. Besides its well documented function in cell-cell adhesion on the paracellular space [36], occludin has been described as an HCV receptor [37]. Human occludin and CD81 expression are sufficient to render murine cells permissive to HCVpp, therefore representing the minimal human-specific entry factors necessary for HCV entry. Moreover, silencing occludin in permissive cells impairs both HCVpp and HCVcc entry [37]. The mechanism whereby ADRP increases the expression of occludin warrants further investigation. One possibility could be that the increase of lipids induced by ADRP overexpression could impact occludin expression. Indeed, a study reported that occludin expression was regulated by polyunsaturated fatty acids [38]. On the other hand, FAS, a key enzyme in neolipogenesis, has been suggested to promote HCV entry by upregulating claudin-1 [39], further hinting at a link between lipid metabolism and tight junctions. Nevertheless, we cannot fully exclude that the increase of HCV entry is due to a mechanism independent of occludin. As ADRP also modifies the level of cellular cholesterol, it is possible that its recruitment to the plasma membrane is increased as well, which could modulate HCV entry efficacy. In fact, it has been shown that the cholesterol depletion using a cholesterol-depleting drug decreases HCV entry [40] and conversely that HCVpp entry and fusion are facilitated when a large amount of cholesterol is present in the target membranes. Finally, our observation that the intracellular HCV RNA level, the core protein level and intracellular particle infectivity are decreased while, at the same time, the number of secreted infectious particles is augmented is an argument in favor of an activation of viral particle egress by ADRP overexpression.

We showed also that HCV increased occludin expression *via* a mechanism specifically dependent on ADRP, since silencing ADRP fully prevented HCV-associated occludin increase. This effect was confirmed by the correlation observed in HCV-infected human livers between occludin and ADRP mRNA’s. The fact that HCV induces one of its receptors may appear counterintuitive, as viruses often establish strategies to downregulate their receptors in order to prevent superinfection [41, 42]. Among *Flaviviridae*, mechanisms of superinfection exclusion have been identified at various steps of the viral life cycle, including entry and replication [43, 44]. Regarding HCV entry, the picture seems complex as conflicting data have been reported. In HCV-infected livers, while no significant changes of CD81 and SR-BI expression were observed when compared to control uninfected tissues, claudin-1 and occludin expression was shown to be significantly increased at the hepatocyte surface [45, 46]. In addition, hepatitis C recurrence after liver transplantation is associated with an increase of claudin-1 and occludin expression at the hepatocyte cell membrane [47]. In cell models, claudin-1 has been shown to be either down- [48] or up-regulated [45]. Other studies have shown that occludin expression was downregulated [48] and that the HCV E2 envelope protein induced the sequestration of occludin in the endoplasmic reticulum, therefore impairing its proper localization to the plasma membrane [49]. One way to reconcile these data is that the expression of HCV receptors could be tightly regulated in a dynamic manner over time, being upregulated at earlier time points after infection in order to promote infection and eventually downregulated to prevent superinfection. In our model, in addition, we cannot rule out the possibility that HCV may increase occludin expression in neighboring uninfected cells *via* paracrine mechanisms to promote viral spread, as suggested by observations performed on claudin-1, the expression of which was increased in NS5A-negative cells within a population of infected cells [45].

Finally, we must mention the fact that the expression of both ADRP [50] and occludin [51] correlates with the degree of steatosis in patients with non-alcoholic fatty liver disease, and that occludin is increased also in hepatocellular carcinoma tissue compared to normal livers [52]. Thus, it is tempting to speculate that the ADRP-occludin axis may provide a working hypothesis for the increased risk of hepatocellular carcinoma in patients with hepatitis C and steatosis [53, 54].

## Conclusion

In conclusion, we show that the upregulation of ADRP by HCV leads to the establishment of an environment favorable to the HCV life cycle, in particular resulting into the promotion of viral spread. This effect is brought about by (i) increasing the overall LD membrane area, thus increasing the surface available for viral particle assembly; (ii) promoting viral egress from infected hepatocytes and (iii) enhancing the virion entry *via* an overexpression of occludin, one of the key viral receptors.

## Supporting Information

S1 Fig Characterization of ADRP-overexpressing or -silenced Huh-7 cells. Huh-7 cells were transduced with a lentivector coding for ADRP. After 48 h, ADRP expression was assessed by either RT-qPCR **(A)** or immunofluorescence **(B)**. Anti-ADRP immunofluorescence of control (a) and ADRP transduced cells (b). **(C)** Representative optical confocal immunofluorescence section of ADRP-overexpressing cells double-stained with ORO (a) and anti-ADRP (b). Nuclei were counterstained with DAPI (c). Merged image is shown in (d). **(D-F)** Huh-7 cells were transfected with siADRP. After 72h, ADRP expression was assessed by either RT-qPCR **(D)** or immunoblotting **(E,F)**.
(PDF)

S1 Table Reagents, antibodies plasmids, primers, and siRNAs. (DOCX)
