# Supplementary figures and images for: Hepatitis C Virus Increases Occludin Expression via the Upregulation of Adipose Differentiation-Related Protein
Source: PLoS One. 2016 Jan 5;11(1):e0146000. doi: 10.1371/journal.pone.0146000 (PMC4701191; doi:10.1371/journal.pone.0146000)

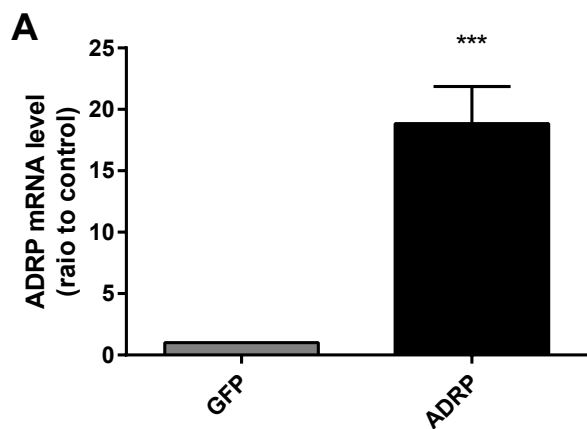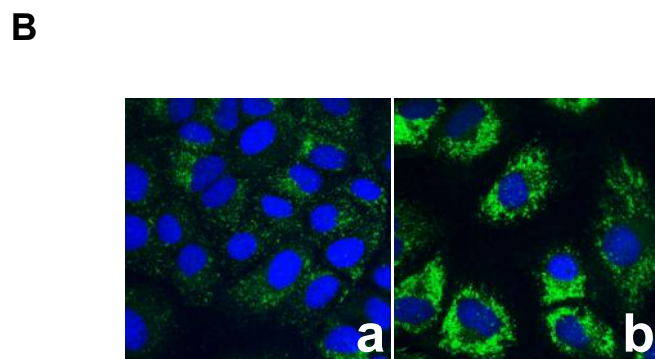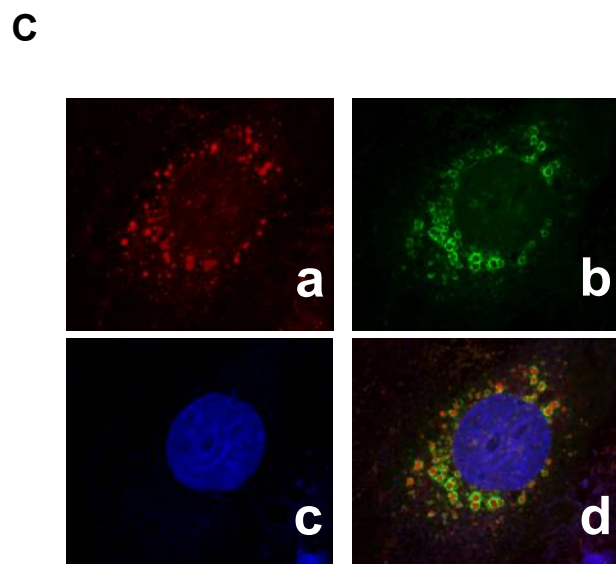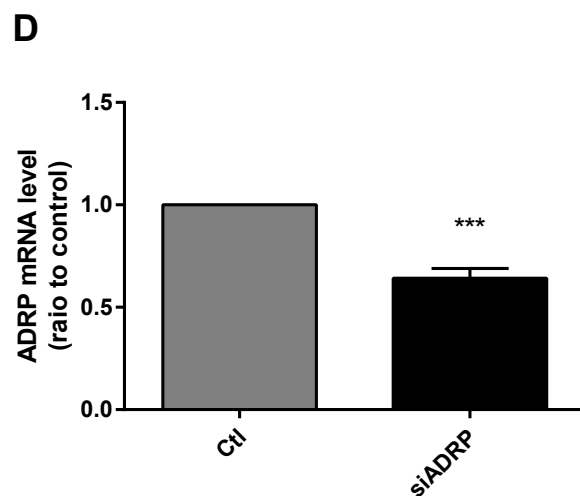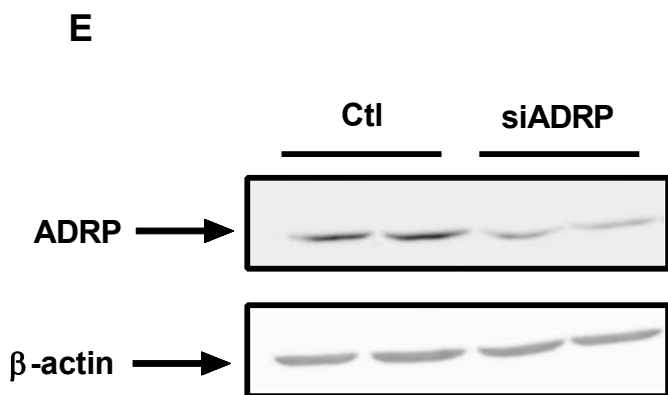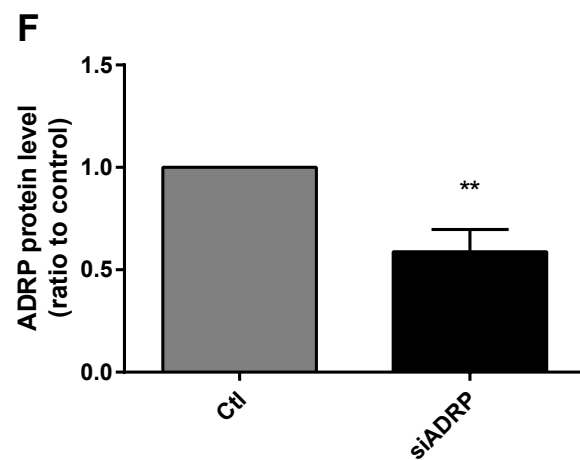

Supplement: S1 Fig — Huh-7 cells were transduced with a lentivector coding for ADRP. After 48 h, ADRP expression was assessed by either RT-qPCR (A) or immunofluorescence (B). Anti-ADRP immunofluorescence of control (a) and ADRP transduced cells (b). (C) Representative optical confocal immunofluorescence section of ADRP-overexpressing cells double-stained with ORO (a) and anti-ADRP (b). Nuclei were counterstained with DAPI (c). Merged image is shown in (d). (D-F) Huh-7 cells were transfected with siADRP. After 72h, ADRP expression was assessed by either RT-qPCR (D) or immunoblotting (E,F). (PDF) [file pone.0146000.s001.pdf]
