# Supplementary material for: Hepatitis C Virus Increases Occludin Expression via the Upregulation of Adipose Differentiation-Related Protein
Source: PLoS One. 2016 Jan 5;11(1):e0146000. doi: 10.1371/journal.pone.0146000 (PMC4701191; doi:10.1371/journal.pone.0146000)
Supplement: S1 Table — (DOCX) [file pone.0146000.s002.docx]

**Hepatitis C Virus Increases Occludin Expression *via* the Upregulation of Adipose Differentiation-Related Protein**

Emilie Branche, Stéphanie Conzelmann, Clotilde Parisot, Ludmila Bedert, Pierre L Lévy, Birke Bartosch, Sophie Clément, and Francesco Negro.

# Supplementary informations

**S1Table: Reagents, antibodies plasmids, primers, and siRNAs used in the study**

**Real-time PCR primers:**

| **Name** | **Forward** | **Reverse** |
| --- | --- | --- |
| Human ADRP | ACC AGT GCT CTG CCC ATC A | CCC CTT ACA GGC ATA GGT ATT GG |
| Human TIP47 | CAG GAA CAG AGC TAC TTC G | CAG TTT CCA TCA GGC TTA GG |
| Human PLIN5 | TTT TGG TAT TGG ACT CTC CCA TTT | CTC CCC CGG GCC TCT T |
| Human PLIN1 | GAC CTC CCT GAG CAG GAG AAT | GTG GGC TTC CTT AGT GCT GG |
| Human SCRAB1 | TGC ACC CTA ACC AGG AGG CAC A | TGC AAA CCA GAG CAG CGG CA |
| Human LDL-R | TGG CCC AGC GAA GAT GCG AA | AGC CTT GCA GGC CTT CGT GT |
| Human CD81 | ACG CCA ACA ACG CCA AGG CT | TGC TGC CCG AGG GAC ACA AA |
| Human CLDN | AAT GCC CGT GGC AAT GGT GG | TCC CGG AAA CCA CAA TAG CGG GA |
| Human OCLN | ACG TGC CTT CAC CCC CAT CTG A | ACC GCT GCT GTA ACG AGGG CT |
| Human NPC1 | AAC AAC CGC ACG CTC CTG CT | ATC CTT GAA GGT GAG CGG GGC A |
| Human EEF1A1 | AGC AAA AAT GAC CCA CCA ATG | GGC CTG GAT GGT TCA GGA TA |
| Human CD36 | AGT CAC TGC GAC ATG ATT AAT GGT | CTG CAA TAC CTG GCT TTT TCT CA |
| Human FATP5 | TCA TCC TGG GCC TGT GTG A | CAT TTC CAG CCC ACT GAG GTT |
| Human ACCalpha | Qiagen QT00033761 | |
| Human FAS | ACA CCC AAG GCC AAG TAC CA | TAG GCG CCA CCC GTC TT |
| Human SREBP-1c | TCA GCG AGG CGG CTT TGG AGC AG | CAT GTC TTC GAT GTC GGT CAG |
| Human HMG-CoA synthase | GTA TGC CCT GGT AGT TGC AGG AG | TGT TGC ATA TGT GTC CCA CGA A |
| Human ACAT | GGG CTA ACT GAT GTC TAC AAT AAA ATT CA | TCG TGC CAA TAT TCA GCT TCTTTG |
| Human SREBP-2 | CCA AGG CCC TGG AAG TGA | GCA TGG AAG ATG GCC TTC A |
| Human PPARalpha | AAT TTG GCT CTT CTG AGG TCA TTA TTA T | CCT AAC CCT AAC TTT CAC TTT TTT CTA GTG |
| Human ApoB | Qiagen QT00020139 | |
| Human ApoE | Qiagen QT00087297 | |
| Human MTP | CAA ACT CAA AGC AGT AGT GG | TGT TTA AGG TCT TCT TCA CCT C |
| PLV2 | ACC TGA AAG CGA AAG GGA AAC | CAC CAA TCT CTC TCC TTC TAG CC |
| HCV | Primers KK30 and KM3 [1] | |

**Primary antibodies:**

| **Protein targeted** | **Host** | **Clone** | **Provider** | **Catalogue number** |
| --- | --- | --- | --- | --- |
| ADRP | Mouse | AP125 | Progen | 610102 |
| OCCLUDIN | Mouse | OC-3F10 | Invitrogen | 331500 |
| HCV core | Mouse | C7-50 | Axxora | ALX-804-277 |
| β-cytoplasmic actin | Mouse | C4 | Chemicon | MAB1501 R |

**Secondary antibodies:**

| **Protein targeted** | **Host** | **Provider** | **Catalogue number** |
| --- | --- | --- | --- |
| HRP-conjugated anti-mouse | Goat | Biorad (Switzerland) | 170-6516 |
| Alexa Fluo 488 anti-mouse | Goat | Life technologies | A11029 |

**Plasmids and HCV constructs:**

| **Plasmid insert/name** | **Backbone** | **Source** | **references** |
| --- | --- | --- | --- |
| ADRP | 2K7 |  |  |
| GFP | 2K7 |  |  |
| OTB-ADRP plasmid |  | Openbiosystem | MHS4771-99610778 |
| pFK-J6/C3 (Jc1) |  | R. Bartenschlager | [2] |
| Core of Jc1 | 2K7 |  |  |
| pFK_i389LucNS3 |  | R.Bartenschlager, Heidelberg, Germany | [3] |
| pTKrenilla_Luc |  | D.Garcin, Geneva, Switzerland |  |

**siRNA**

| **Name** | **Provider** | **Catalogue number** |
| --- | --- | --- |
| ADRP siRNA | Qiagen | SI02780043 |
| Control siRNA | Cell Signaling | 6568 |

**Other reagents**

| **Name** | **Provider** | **Catalogue number** |
| --- | --- | --- |
| Nucleospin RNA II | Macherey-Nagel (Germany) | 740.955.50 |
| Random hexamer primers | Invitrogen (CA, USA) | 48190-011 |
| SuperScript II Reverse Transcriptase | Invitrogen (CA, USA) | 18064-022 |
| QIAamp Viral RNA Mini Kit | Qiagen (Switzerland) | 52904 |
| Oil Red O | Sigma (MO, USA) | 75087 |
| Triglyceride kit | Roche/Hitachi (Germany) | 12016648-122 |
| Cholesterol/cholesteryl ester quantitation kit | Calbiochem (Germany) | 428901-1 |
| BCA protein Assay kit | Pierce | 23227 |
| Dual-Luciferase assay system kit | Promega | E1910 |
| Trizol | Ambion | 15596018 |
| Lipofectamine 2000 | ThermoFischer  Scientific | 11668-019 |
| Amaxa cell line nucleofector kit | Lonza | VCA-1002 |

# References

1. Takeuchi T, Katsume A, Tanaka T, et al. Real-time detection system for quantification of hepatitis C virus genome. Gastroenterology 1999;**116**(3):636-42

2. Barba G, Harper F, Harada T, et al. Hepatitis C virus core protein shows a cytoplasmic localization and associates to cellular lipid storage droplets. Proc Natl Acad Sci U S A 1997;**94**(4):1200-5

3. Lohmann V, Hoffmann S, Herian U, Penin F, Bartenschlager R. Viral and cellular determinants of hepatitis C virus RNA replication in cell culture. J Virol 2003;**77**(5):3007-19
